# The Pathogenic Role of Dysregulated Epigenetic Modifications in Autoimmune Diseases

**DOI:** 10.3389/fimmu.2019.02305

**Published:** 2019-09-27

**Authors:** Haijing Wu, Yongjian Chen, Huan Zhu, Ming Zhao, Qianjin Lu

**Affiliations:** Hunan Key Laboratory of Medical Epigenomics, Department of Dermatology, Second Xiangya Hospital, Central South University, Changsha, China

**Keywords:** epigenetics, autoimmunity, SLE, DNA methylation, miRNAs

## Abstract

Autoimmune diseases can be chronic with relapse of inflammatory symptoms, but it can be also acute and life-threatening if immune cells destroy life-supporting organs, such as lupus nephritis. The etiopathogenesis of autoimmune diseases has been revealed as that genetics and environmental factors-mediated dysregulated immune responses contribute to the initiation and development of autoimmune disorders. However, the current understanding of pathogenesis is limited and the underlying mechanism has not been well defined, which lows the development of novel biomarkers and new therapeutic strategies for autoimmune diseases. To improve this, broadening and deepening our understanding of pathogenesis is an unmet need. As genetic susceptibility cannot explain the low accordance rate of incidence in homozygous twins, epigenetic regulations might be an additional explanation. Therefore, this review will summarize current progress of studies on epigenetic dysregulations contributing to autoimmune diseases, including SLE, rheumatoid arthritis (RA), psoriasis, type 1 diabetes (T1D), and systemic sclerosis (SSc), hopefully providing opinions on orientation of future research, as well as discussing the clinical utilization of potential biomarkers and therapeutic strategies for these diseases.

## Introduction

Autoimmunity is a pathological condition that self-immune system cannot distinguish self-antigens and attacks self-tissues and organs, resulting in inflammation and organ damages. Autoimmune diseases, such as SLE, RA, and T1D are sometimes referred to as “invisible disabilities” or life-threatening diseases, with high incidence rate of 11%. Approximately 600 million people suffer from a breakdown of immune tolerance. Aberrant differentiation and function of immune cells are believed to be a key player in the pathogenesis of autoimmune diseases. However, the molecular mechanism remains unknown.

Genetic susceptibility can partially explain some of the abnormalities of immune imbalance. For instance, over 60 genes have been revealed in previous genetic studies as risk genes in lupus, and some of them have been found to be related to antibody production, complementary deficiency and renal involvements (1). However, genetic studies cannot completely explain the incidence rate of SLE in homozygous twins ranges from 24 to 58% (2), indicating that in addition to genetics, environment factors are also involved in the pathogenesis of SLE. As one of molecular mechanisms of environmental factors, epigenetics has been proposed as a critical player in the diseases by accumulating evidence, and it might provide additional explanation for the dysregulation of immune system. Therefore, this review focuses on the current understanding of epigenetic-mediated regulations on immune cell differentiation and functions, summarizes the contribution of dysregulated epigenetic modifications to autoimmune disorders, and discusses the possibility of utilization of unique and specific epigenetic modifications as potential biomarkers and novel therapeutic targets for these diseases.

## Epigenetics in Pathogenesis of Autoimmune Diseases

All cells and tissues in our body share the same set of genomic DNA, however, cells display various morphology and phenotypes due to the gene transcription mediated by epigenetics. Epigenetics is a biological process that recruits or removes reversible and potentially heritable modifications in genomic DNA and/or chromatin but does not change DNA sequence. It is mainly comprised of DNA methylation, histone modifications, and non-coding RNAs-mediated regulations. Epigenetic regulations participate in numerous biological process, such as cell proliferation and differentiation. and increasing evidence has shown that dysregulated epigenetic modifications are involved in pathogenesis of several autoimmune diseases (3–6). The influence of environmental factors, such as UVB, and disease predominance in female emphasizing the importance of epigenetics in the pathogenesis of autoimmune disorders (7). In addition, 5-azacytidine and procainamide (8) are capable of inducing lupus via epigenetic alterations. Similar phenomena have been found in other autoimmune diseases: dysregulation of epigenetic modifications in RA synovial fibroblasts (RASF) leading to abnormal gene expression (9), Epstein-Barr virus (EBV) infection, sunlight (10, 11) and aberrantly expressed miRNAs (12, 13) contributing to the pathogenesis of multiple sclerosis (MS).

As the intensively studied epigenetic modification, DNA methylation refers to a well-known biological process which involves a recruitment of a methyl group to a cytosine or adenine residue at the 5th position on the pyrimidine ring, resulting in inhibiting the binding of transcription factors on the promoter region of gene, which will repress the gene transcription (14). This process is mainly regulated by methyltransferase, including DNA methyltransferase 1 (DNMT1), DNMT3a, and DNMT3b. Each of methyltransferase executes different functions. For instance, during cell replication DNMT1 maintains the methylation levels, whereas DNMT3a and DNMT3b promote methylation process (15). On the contrary, DNA hydroxymethylation, and demethylation are processes that re-activate transcription of silenced genes (16). DNA hydroxymethylation is an instable status and in the middle of demethylation process. DNA hydroxymethylation is mediated by hydroxymethylation transferases, such as ten-eleven translocation methylcytosine dioxygenase 1 (TET1), TET2, and TET3 (17).

Histone modification is a covalent post-translational regulation that modulates gene transcription by altering the structure of chromatin. Histone modifications include methylation, acetylation, ubiquitination, phosphorylation, sumoylation, etc. (18). Acetylation and deacetylation are intensively studied ones which can recruit or remove an acetyl group on histones, thereby activating, or inhibiting gene transcription. Mechanically, acetylation activates gene transcription by opening the chromatin structure and facilitating the binding of transcription factors, while methylation converts opened chromatins into a restrictive structure, inhibiting the binding of transcription factors via stereo hindrance, thereby resulting in the repression of gene expression. Acetylation is mediated by histone acetyltransferases (HATs), deacetylation is regulated by histone deacetylases (HDACs) (19). However, the effects of histone modifications vary depending on the modification positions and the number of modifications. For example, H3K4me3 promotes gene expression whereas H3K9me3 and H3K27me3 represses gene transcription (20, 21).

microRNAs (miRNAs) are small non-coding RNAs, which are usually 21-25 base pairs. It has been well established that miRNAs modulate gene expression at posttranscriptional and posttranslational level through binding to the 3′-UTRs of target mRNAs, resulting in blocking gene translation by mRNA cleavage and degradation (22–24). Besides, long ncRNAs are recently identified non-coding RNAs, with the length of >200 nt. Differing from miRNAs, lncRNAs can either promote or inhibit gene expression. LncRNAs usually act by complexes of lncRNA: RNA, lncRNA: protein or lncRNA: chromatin (25, 26). Accumulating evidence suggests that lncRNAs are involved in numerous human diseases, such as cancer, by changing the primary and secondary structure of DNA, thereby regulating gene expression (27, 28).

## Dysregulated Epigenetic Regulations in Autoimmune Diseases

### Abnormal DNA Methylation in Autoimmune Diseases

#### DNA Hypomethylation in SLE

SLE is a multi-organ involved autoimmune disease that is characterized by aberrant immune cells, such as dendritic cells, B and T lymphocytes. Although the pathogenesis of SLE has been studied for over a century, the exact cause of lupus remains unknown. Increasing evidence from varies groups, including our group, have reported that DNA methylation plays a critical role in immune cells hyper-active in lupus conditions.

##### DNA hypomethylation in lupus T cells

The first evidence of epigenetic regulation in lupus is from the observation that after a long-term administration of two DNA methylation inhibitors, procainamide and hydralazine, normal mice showed a lupus-like phenotype. In addition, cells from thymus and lymph nodules from MRL/lpr mice (spontaneous lupus mouse model) show lower DNA methylation level compared with cells from MRL/mpj control mice (29, 30). This evidence might provide an explanation for over-proliferated and over-activated immune cells in lupus mice.

Lupus T cell auto-reactivity is found to attribute to DNA hypomethylation (31). These findings were further confirmed by the evidence that induction of auto-reactive CD4^+^ T cells from healthy controls by the administration of 5-azacytidine (31, 32), which followed prior evidence of the induction of IL-2 and IFN-γ by the same drug (33). Accumulating evidence have revealed the regulatory effects of DNA methylation on individual genes during the T cell activation and differentiation. IFN-γ and IL-4 are signature cytokines for Th1 and Th2 program, respectively. During Th1 and Th2 differentiation processes, DNA hypomethylation level has been observed at *Ifng* and *Il4* loci (34, 35). In addition, compared to naïve T cells, decreased DNA methylation level is found at the key transcription factor FOXP3 locus in regulatory T cells (Treg) (36). Furthermore, the key transcription factor Bcl6 in Tfh cell has been reported to be highly expressed but with a decreased level of 5hmC (37) during Tfh cell differentiation, suggesting that Tfh cell differentiation is also mediated by DNA methylation modification.

In addition, genomic DNA in lupus CD4^+^ T cells has been found to show DNA hypomethylation (38, 39). DNA hypomethylation has been observed on promoter region of *lfa-1* in CD4^+^ T cells from active lupus patients and over-expressed LFA-1 has been found on an autoreactive subset of T cells, which produces perforin and granzyme B to lyse autologous cells (31, 40), thereby inducing inflammation and tissue damages. Epigenetic accessibility and transcriptional poising of interferon-regulated genes in Naïve CD4^+^ T cells from SLE patients have been shown in a genome-wide DNA methylation study (41). In this study, DNA hypomethylation is observed on interferon-regulated genes, such as IFI44L, which suggest that lupus T cell progenitors have abnormalities (41). More interesting is that our recent studies have proposed DNA hypomethylation level on IFI44L promoter as a biomarker for the diagnosis of lupus, which have both high sensitivity and specificity (42). In a consequent study, different DNA methylation patterns have been observed in organ-specific manner in lupus. For instance, different DNA methylation patterns have been on lupus patients with renal involvement vs. non-renal involvements, and malar rash vs. discoid rash (43). Interesting, some protein such as RFX1 (44), high mobility group box protein 1(HMGB1) (45) and DNA Damage-Inducible 45 alpha (Gadd45a) (46) have been revealed as regulators for this epigenetic regulation by our previous studies.

Besides, in lupus CD4^+^ T cells, 5-hmC binds in transcriptional regulatory regions of lineage-specific signature genes, such as IL-17 and IFN-gamma, which promote inflammation. Mechanically, TET2 protein, a hydroxymethylation transferase, is found to be recruited to 5-hmC-binding regions of *Il17* and *Ifnr*, and then promotes the production of IL-17 and IFN-gamma (47). We have recently observed that lupus CD4^+^ T cells display an increased 5-hmC level on whole genomic DNA compared with normal controls, with the enhanced expression of TET2 and TET3. As a consequence of DNA demethylation, transcription activator CTCF binds to the promoter region of SOCS1 and therefore promotes SOCS1 over-expression in SLE CD4^+^ T cells (48).

##### DNA hypomethylation in lupus B cells

SLE is an autoantibody-mediated autoimmune disorder. As the main and unique origin of autoantibodies, numerous evidence has well document that B cell plays an essential role in the pathogenesis of SLE. Pre-clinical studies and clinical trials of B cell-targeting treatments have proven to be effective to some extent. Not to our surprise, DNA hypomethylation has been also shown in lupus B cells (49), which might regulate B cell development, differentiation, and auto-reactivity. For example, abnormally expressed HRES1/p28 by lupus B cells is reported to be regulated via DNA methylation (50). DNA hypomethylation on *LINE1* gene has been shown in lupus B cells (51). The regulatory effect of DNA methylation in B cells is further supported by the evidence that enhanced levels of anti-nuclear antibodies can be induced by adoptive transferring of DNMT1 inhibitor-treated B cells (52). Although it is elucidated that antibody production is attributed to DNA hypomethylation in V(D)J region and Igh 3′-LCR (53), little has been revealed in this process in the lupus condition. Furthermore, in auto-reactive B cells, DNA hypomethylation might be a result of decreased level of DNMT1 and DNMT3b, or active DNA demethylation mediated by activation-induced cytidine deaminase (AID) (54).

#### Aberrant DNA Methylation in Psoriasis

Psoriasis is a chronic inflammatory autoimmune skin disease, which is characterized by hyper proliferation of keratinocytes and dysregulated T cells, especially Th17 cells (55). Similar with SLE, genetic susceptibility is not the only factor for the onset of this disease, due to that the concordance of psoriasis in monozygotic twins is 35–72% (56), suggesting that epigenetic regulations might be an additional factor. Increased evidence has shown the critical role of DNA methylation in the hyper-proliferated keratinocytes.

In our previous study, abnormal DNA methylation pattern has been observed in skin lesions and PBMCs of patients with psoriasis vulgaris (57, 58). On the gene specific level, the abnormal methylation pattern on the promoter of *p16*^INK4a^ gene has been reported in psoriatic epidermis (59). Increased DNA methylation level on promotor of secreted frizzled-related protein (*Sfrp4*) has been observed in inflamed psoriatic skin and in the IL-23-induced psoriatic mice, thereby reducing the expression of *Sfrp4*, a negative regulator for keratinocyte proliferation (60). Hypomethylation of *LINE-1* has been found in psoriatic keratinocytes. More importantly, manipulating *LINE-1* methylation may change the gene expression, thereby resulting in a phenotypic alteration of psoriatic skin (61). In addition, aberrant DNA methylation pattern has also been revealed in CD4^+^ T cells from psoriatic patients (62), indicating that the epigenetic regulations on immune cells also attributing to psoriasis pathogenesis.

#### Aberrant DNA Methylation Status in RA

RA is an autoreactive immune cell-mediated inflammation which primarily affects joints. Autoreactive immune cells and synovial fibroblasts (SF) are well defined as the critical players in the pathogenesis of RA. Heterogeneity in RA patients is a hindrance for rheumatologists and dermatologists to diagnose and treat patients. The treatment of RA is always delayed due to the current criteria that in addition to meeting all diagnostic criteria, RA patients need to consistently display arthritic symptoms for at least 6 months (63). Early intervention is necessary because a clinical trial on BeSt have shown that BeSt can delay the onset of RA on several patients (64).

Increasing evidence has shown that DNA methylation contributes to the pathogenesis of RA. Increased DNA methylation variability has been observed in rheumatoid arthritis-discordant monozygotic twins (65), indicating the importance of DNA methylation in the pathogenesis of RA. Abnormal genome-wide DNA methylation patterns have been revealed in CD4^+^ T cells from Chinese Han patients with rheumatoid arthritis (66). In PBMCs from RA patients, decreased DNA methylation levels have been found at the promoter regions of *Il6* and *ERa*, which may be associated with over-production of IL-6 and hyperactive ERa signaling (67–69). Global DNA hypomethylation is also found in T cells from RA patients (31, 70). On the gene specific level, *CD40L* gene has found to be demethylated on CD4^+^ T cells from RA patients (69). Moreover, DNA hypomethylation on promoter region of *L1 retrotransposon* gene has been observed in RA fibroblast-like synoviocytes (71, 72). Further, DNA hypomethylation on *CXCL12* gene has been shown in synovial fibroblasts, that may result in cell infiltration in joints (73, 74). More interesting, DNA methylation status has been proposed as biomarkers to predict the drug responses (75).

#### Dysregulated DNA Methylation in Systemic Sclerosis (SSc)

SSc is a relatively rare disease which is characterized by damages of connective tissues mediated by autoreactive immune cells. Its etiopathogenesis remains unclear. Abnormal epigenetic modifications have been shown in SSc. In an integration study of Genome-Wide DNA Methylation and Transcription, several DNA methylation regulated-gene expression have been revealed in SSc PBMCs (76) and dermal fibroblasts (77). Decreased DNA methylation level has been observed in CD4^+^ T cells from SSc patients and reduced expression of DNMTs have been found in CD4^+^ T cells from these patients (78). DNA demethylation on promoter regions of *CD11a, CD70*, and *CD40L* genes have been found in CD4^+^ T cells from SSc patients (78–81). However, hypermethylated genes, such as *PRF1, CDKN2A, Foxp3, CD11a*, and *CD70*, have been observed in whole blood from black South African patients with SSc (82). Moreover, as the key transcription factor to Th17 cells, *RORC1* and *RORC2* have been found to show hypomethylation and be correlated with inflammatory status in SSc PBMCs (83). Furthermore, in dermal fibroblasts from SSc patients, hypermethylation has been found in *FLl1* and TGF-beta-related genes, which are Wnt pathway antagonist genes (84–86), accompanied by increased levels of DNMT1 (87) and TET1 (88).

#### Abnormal DNA Methylation Levels in T1D

T1D is well-documented as an autoimmune disease, which is mainly mediated by T cells by attacking beta cells. In an epigenome-wide association study (GWAS) in 52 monozygotic twins, epigenetic modification patterns have been mapped in CD4^+^ T cells, CD19^+^ B cells, and CD14^+^ monocytes (89). This study has identified a substantial enrichment of differentially variable CpG positions (89), suggesting the involvement of DNA methylation in T1D. In addition, differential DNA methylation status on 88 CpG sites has been found in lymphoblast cell lines which are derived from 6 pairs of monozygotic twins concordant for T1D and 3 pairs of monozygotic twins discordant for T1D, separately. In these cells lines, the altered expression of genes, including *Hla, Ins* and *Il2rb*, are involved in immune responses (90). Furthermore, dysregulated DNA methylation have been found in *Pdchb16, Magi2*, and *Fancc* in T1D-discordant monozygotic twins (91). DNA demethylation on transcription factor *HOXA9* has been observed in T1D patients (92). DNA hypermethylation has been found in the promoter region of *Foxp3*, which represses the binding of transcription factor *IRF-7* to *Foxp3*, resulting in the reduced number of regulatory T cells in the peripheral blood from T1D patients (93). More interesting, the serum levels of unmethylated preproinsulin DNA might serve as a biomarker for T1D (91). Dysregulated DNA methylation are listed in Table 1.

**Table 1:** Dysregulated DNA methylation in autoimmune diseases: SLE, Psoriasis, RA, SSc, and T1D.

**Disease**	**Origens**	**DNA methylation status**	**References**
SLE	Whole blood	*IFI44L*: hypomethylation FOXP3 TSDR: hypermethylation	(48) (42)
SLE	PBMCs	Global, *ERa*: hypomethylation	(38, 67)
SLE	T cells	X chromosome genes, *IL4, IL6:* hypomethylation	(94–96)
SLE	CD4^+^ T cells	Global, IFN-regulated genes, *perforin, PP2Aca, KIR2DL4, CD11a, CD70, CD40L, IL10, IL13:* hypomethylation	(97–104)
SLE	Naïve CD4^+^ T cells	IFN-regulated genes, *MIR886, TRIM69, CHST12*: hypomethylation	(41, 43, 105)
SLE	B cell	IFN-regulated genes: hypomethylation *LINE-1:* hypomethylation	(98) (51)
SLE	Monocytes	IFN-regulated genes: hypomethylation	(98)
Psoriasis	PBMCs, skin lesion	Aberrant DNA methylation pattern	(57, 58)
Psoriasis	Keratinocytes	p16INK4a: abnormal DNA methylation level *Sfrp4: hypermethylation LINE-1: hypomethlation*	(59) (60) (61)
Psoriasis	CD4^+^ T cells	Aberrant DNA methylation pattern	(62)
RA	PBMCs	*IL6, ERa*: hypomethylation	(67–69)
RA	T cells	Global: hypomethylation	(31, 70)
RA	CD4^+^ T cells	*CD40L*: hypomethylation	(69)
RA	Fibroblast-like synoviocytes	Global, *L1 retrotransposon*: hypomethylation	(71, 72)
RA	Synovial fibroblasts	Global, *CXCL12*: hypomethylation	(73, 74)
SSc	CD4^+^ T cells	Global, *CD40L CD11a, CD70*: hypomethylation	(78–81)
SSc	Dermal fibroblasts	*FLl1*, TGF-beta-related genes: hypermethylation	(84–86)
T1D	PBMCs	*HOXA9*: hypomethylation	(92)
T1D	Treg cells	*Foxp3*: hypermethylation	(93)

### Aberrant Histone Modifications in Autoimmune Diseases

#### Dysregulated Histone Modifications in Lupus

Lupus CD4^+^ T cells show global histone H3 and H4 hypoacetylation (106). Abnormal histone modifications have been found in the promoter region of *TNFSF7* in T cells, resulting in overexpression of CD70, which might be the one cause of auto-reactivity of T cells (107). Administrating HDAC inhibitors on healthy T cells results in decreased CD3ς chain expression, thereby leading abnormalities in T cells (108). A transcription factor CREMα might be involved in the process of histone acetylation in active lupus T cells via inhibition of IL-2 production. This process might be mediated by recruiting HDAC to *Cre* binding sites in the promoter region of *Il2* (109). Besides, abnormal H3K4me3 modification has been observed on lupus-related candidate genes in lupus PBMCs (110). Lupus monocytes show altered acetylation status of global H4. Among them, 63% of these H4 acetylated genes are potentially modulated by IFN regulatory factors (111), which are involved in the pathogenesis of SLE.

In addition to the whole genomic modifications, histone modification has been reported to modify specific gene expression. For example, increased H3 acetylation level has been found at the IL-17 locus and enhanced IL-10 production has been revealed to be mediated by chromatin remodeling. This process is further revealed to be mediated by Stat3 (112, 113). Moreover, histone hyperacetylation has been shown to be a cause for an increased serum level of TNF-α and an enhanced maturation status of monocytes from lupus patients (114). However, it is still unclear whether histone modifications are the initiator or results of immune disorders, even though the contribution of histone modifications in pathogenesis of lupus has been revealed in mouse studies.

Sirtuin-1 (Sirt-1) is a histone deacetylase, which has been observed to be overexpressed by T cells from MRL/lpr mice (115). Knocking down Sirt-1 in lupus mice leads to a temporary enhancement of H3 and H4 acetylation, accompanied by attenuated lupus symptoms such as reduced serum levels of anti-dsDNA, IgG deposition in glomerular and histological changes (38). Treating MRL/lpr mice with HDAC inhibitors can attenuate renal damage and decrease level of inflammatory cytokines (116). A recent progress has been made from a genetic and epigenetic mapping study which identifies candidate causal variants in 21 autoimmune diseases in different T cell subtypes, including Th1, Th2, Treg, and Th17 cells (117). In this study, unique H3K27 peaks are shown in the super-enhancer in *Il2RA* locus, particularly in Treg and Th17 cells.

In our previous study, we have demonstrated that RFX1 inhibits Th17 cell differentiation via increased histone H3 acetylation, decreased DNA methylation and H3K9 tri-methylation (118), thereby contributing to SLE pathogenesis. More recently, the downregulation of TNF-alpha-induced protein 3 (TNFAIP3), one of the major SLE susceptibility genes involving in the regulation of inflammatory responses through modulation of the nuclear factor-kappaB (NF-kappaB) pathway, has been observed in lupus patients. This downregulation may be mediated by reduced H3K4me3 in the gene promotor region (119), providing a promising target for the treatment of SLE in clinical practice.

In addition, some epigenetic targeting therapies also revealed the importance of histone modification in pathogenesis of autoimmune diseases. For example, selective HDAC6 inhibition has been shown to attenuate early stage of lupus nephritis via down-regulation both innate and adaptive immune responses (120). Selective HDAC6 inhibitor also showed therapeutic effects on lupus mice by improving renal function and survival (121). A novel histone deacetylase 3-selective inhibitor has been reported to inhibit IL-6 production by PBMCs from RA patients (122). As well as in T1D, histone deacetylase inhibitors have been shown to modify pancreatic cell fate determination and amplify endocrine progenitors (123).

### Aberrant Non-coding RNA Mediating Regulations in Autoimmune Diseases

#### Aberrant Non-coding RNA Mediating Regulations in Lupus

##### Dysregulated non-coding RNAs in lupus T cells

It has been well-documented that miRNAs can bind to various regions but modulate the same gene expression. A large number of miRNAs have been reported to be aberrantly expressed by T cells. Some of these miRNAs have been found to target lupus-related genes, such as *Il10, Il17*, and *dnmt1*. It has been reported that the expression level of miR-21, miR-126 and miR148a is observed to be reduced in lupus T cells and they are found to target DNMT1, although they bind to different regions of DNMT1(124, 125). Furthermore, the inhibition of miR-21, miR-29b, and miR-148a in SLE T cells has been found to be capable of attenuating lupus phenotypes, suggesting potential therapeutic roles in SLE (125, 126). In addition, miR-21 has been found to inhibit the expression of PDCD4 on lupus T cells, thereby promoting T cell proliferation and the expression of CD40L and IL-10 (127). Moreover, miR-142 (128) and miR-31 (129) have been demonstrate to modulate T cell activity by suppressing IL-4 and IL-10 production by T cells, inhibiting the expression of CD40L and ICOS and enhancing secretion of IL-2 by T cells. In addition to our previous studies on aberrantly expressed miR-146a and−241-3p/5p by lupus T cells, we have further found that mycophenolic acid, which has been commonly utilized in clinic for lupus treatment, attenuates the auto-reactivity of lupus T cells through miR-146a and−241-3p/5p, suggesting the pathogenic role of these two miRNAs in SLE (130).

More recently, in short time-series expression miner analysis, some lncRNAs from lupus T cells have been found to be correlated with SLE disease activity (131), suggesting that the aberrant expression profile of lncRNAs may play a role in SLE pathogenesis. In addition, large intergenic non-coding RNAs (lincRNAs), a specific type of lncRNAs, can also modulate gene expression and is involved in various biological processes and diseases. For example, lupus PBMCs show lower level of linc0597and Linc0949, compared to those from rheumatoid arthritis patients and normal subjects (132). More importantly, the decreased level of linc0949 is correlated with the level of C3, SLE disease activity index (SLEDAI), and the appearance of lupus-specific organ damages. More interesting is that the levels of linc0949 can increase significantly depending on efficiency of treatment in lupus patients, suggesting a role as a biomarker for SLEDAI and drug response (132, 133).

##### Aberrantly expressed microRNAs in lupus B cells

As critical regulators in B cell development and differentiation, miRNAs are also involved in the aberrant B cell expression and functions. Lupus B cells shows increased levels of miR-30a. The level of miR-30a in lupus B cells negatively correlates with Lyn, which negatively regulates B cell activation (134). It has been found that miR-155 and miR-181b negatively regulate AID expression, thereby modulating antibody diversity (135, 136). In lupus-prone mice, the levels of miR-15a in regulatory B cells are positively correlate with the serum level of anti-dsDNA antibodies (137). In our recent studies, increased expression of miR-1246 has been observed in lupus B cells, and it has been found to regulate EBF1 expression, thereby promoting the expression of CD40 and antibody production (138). Moreover, enhanced levels of miR-17-92 and miR-21 have been found in SLE B cells (139, 140). More interesting, miRNA profiling of B cell subsets has been proposed as a biomarker for lupus (141), indicating a critical role of miRNAs in lupus abnormal B cells. Moreover, miR-150 is found to be decreased in B cells from MRL-lpr mice, which might be a result of a decreased acetylation level and inhibition expression of the miR-150 host gene (142). miRNAs that are dysregulated in other autoimmune diseases are summarized in Table 2.

**Table 2:** Dysregulated miRNA expressions in: SLE, Psoriasis, RA, SSc, and T1D.

**Disease**	**Origins**	**Levels of miRNAs**	**Target genes**	**References**
SLE	PBMCs	miR-155: + miR-146a: –	*PP2Ac IFNa and IFNb*	(143, 144)
SLE	T cells	miR-21: + miR-31: –	*PDCD4 RhoA*	(127, 129)
SLE	CD4^+^ T cells	miR-142-3p/5p: – miR-21, 148a, 126 and 29b: +	*SAP, CD84, and Il10 DNMT1*	(124–126, 128)
SLE	B cells	miR-30a: + miR-1246: –	*Lyn EBF1*	(134, 138)
Psoriasis	T cells	mir-210	*STAT3, Lyn*	(145)
	Keratinocytes	mir-17–92 cluster	–	(146)
		Mir-let 7b Mir-194	*Il6 Grainyhead-like 2*	(147) (148)
RA	T cells	miR-223: –	*IGF-1R*	(149)
RA	CD4^+^ T cells	miR-146a: +	*FAF1*	(150)
RA	Synovial fibroblasts	miR-155: +	*MMP-3*	(151, 152)
SSc	Fibroblasts	miR-21: + miR-29a: − miR-196a: +	*Smad7* Type I and III collagen Type I collagen	(153–156)
T1D	Plasma	microRNA-16-5p,−17-5p and−20a-5p: +	—	(157)
T1D	Plasma-derived exosome	miRNAs signature	—	(158)
T1D	Treg	miR-125a-5p: +	*CCR2*	(159)
T1D	Beta cell	microRNA-503: +	*mTOR pathway*	(160)
T1D	Plasma	miRNAs profile, miRNA-320a and mRNA-486	–	(161, 162)
T1D	Urine	miRNAs profile	Predict disease	(163)

*+, Increased; –, Decreased*.

#### Aberrant Micro-RNA Mediating Regulations in Psoriasis

Similar, some abnormally miRNAs have been reported in psoriatic patients. In our previous studies, mir-210 is found to be overexpressed by T cells filtrating in the dermis of psoriatic lesions. Further, mir-210 is capable of inducing helper T (Th) 17 and Th1 cell differentiation but inhibiting Th2 differentiation by repressing expression of STAT6 and LYN (145). In addition, the upstream regulation has been revealed as that TGF-beta and IL-23 enhance miR-210 expression by inducing HIF-1alpha, which recruits P300 and promotes histone H3 acetylation in the miR-210 promoter region (145). As Th17 cells playing a critical role in pathogenesis of psoriasis, targeting mir-210 might provide potential therapeutic strategies for psoriasis patients. Besides, mir-17-92 cluster has been revealed to promotes the proliferation and the chemokine production of keratinocytes (146), mir-let-7b has been shown to inhibit keratinocyte differentiation by targeting IL-6 mediated ERK signaling in psoriasis (147), mir-194 has been demonstrated to regulate keratinocyte proliferation and differentiation via Grainyhead-like 2 in psoriasis (148).

## Conclusion

As the epigenetic era approaches, more and more evidence has shown the importance of epigenetic regulations in the pathogenesis of autoimmune diseases. Newly discovered non-coding RNAs, such as LncRNA, extra RNAs and circle RNAs have begun to undergo significant research into their roles in disease pathogenesis. The specific epigenetic regulations in autoimmune diseases might provide potential biomarkers for diseases. For example, in our previous study, the DNA methylation level of the *IFI44L* promoter is both sensitive and specific in lupus patients and lower in nephritis patients than in patients without renal damage (164), indicating an organ-specific biomarker to predict LN. Another urgent need is to be able to translate research findings into clinical application. The most significant challenges include complex techniques, time consuming, and the high cost of DNA methylation arrays and bisulfite next-generation sequencing. To solve this problem, as in our study of *IFI44L*, rather than pyrosequencing of *IFI44L* DNA methylation levels, we have developed a high-resolution melting (HRM) analysis for detecting *IFI44L* DNA methylation levels, which can be easily completed with QPCR. This new technique may be more available for clinical use in the future. With regard to treatment, as our new finding on miR-210 in mouse psoriasis treatment (145), miRNAs might provide alternative options to currently used drugs. The application of Crisper-Cas9 may shed light by guiding epigenetic modifications on specific genes. Together, epigenetic modifications provide additional tools for broadening the understanding of autoimmune diseases, as well as development of potential biomarkers and therapies.

## Author Contributions

HW and YC wrote the manuscript. HZ edited the manuscript. MZ and QL revised the manuscript.

### Conflict of Interest

The authors declare that the research was conducted in the absence of any commercial or financial relationships that could be construed as a potential conflict of interest.
